# Positieve beoordeling van de zorg weinig veranderd tijdens de coronapandemie, maar nog altijd ruimte voor verbetering

**DOI:** 10.1007/s12508-022-00329-y

**Published:** 2022-01-26

**Authors:** Juliane Menting, Mariska Scheffer, Peter Spreeuwenberg, Femke van Schelven

**Affiliations:** Nederlands Instituut voor Onderzoek van de Gezondheidszorg (Nivel), Utrecht, Nederland

**Keywords:** chronische ziekte, coronapandemie, patient reported experience measures, kwaliteit van zorg, patiënttevredenheid, Chronic disease, COVID-19-pandemic, Patient reported experience measures, Quality of care, Patient satisfaction

## Abstract

**Inleiding:**

Het meten van patiëntervaringen geeft belangrijke inzichten in de kwaliteit van de Nederlandse gezondheidszorg. Het huidige onderzoek toetst in hoeverre de ervaren kwaliteit van zorg door de jaren heen is veranderd en hoe deze samenhangt met veranderingen in zorg en gezondheid tijdens de coronapandemie.

**Methode:**

Patiëntervaringen zijn verzameld met tevredenheidsoordelen en de kwaliteitsindicator PREM Chronische Zorg, onder een representatieve steekproef van mensen met een chronische ziekte. Trendanalyses (2016–2020) zijn uitgevoerd en verschillen tussen subgroepen zijn getoetst met Mann-Whitney U‑toetsen.

**Resultaten:**

De kwaliteit van de zorg wordt over het algemeen positief ervaren, ook tijdens de coronapandemie in het najaar van 2020. In dat jaar zijn mensen het minst tevreden over de afstemming tussen zorgverleners en over de preventieve begeleiding van hun ziekte (respectievelijk 64% en 67% is (helemaal) tevreden). Trendanalyses laten zien dat de tevredenheid over preventieve begeleiding is gedaald en dat de tevredenheid over gezamenlijke besluitvorming door de jaren heen schommelt. Mensen die gevolgen van de coronapandemie ervaren voor hun zorg of gezondheid beoordelen aspecten van de gezondheidszorg minder positief dan diegenen die geen gevolgen ervaren.

**Conclusie:**

Het is belangrijk om aandacht te hebben voor patiëntervaringen met zorgprocessen, waarbij extra nadruk zou moeten liggen op informatie over preventie, ondersteuning bij veranderingen in de gezondheid en de behandeling tijdens de coronapandemie, en goede afstemming tussen zorgverleners.

**Digitaal aanvullende content:**

De online versie van dit artikel (10.1007/s12508-022-00329-y) bevat aanvullend materiaal, toegankelijk voor daartoe geautoriseerde gebruikers.

## Abstract (en)

**Introduction:**

Measuring patient experiences provides important insight into the quality of Dutch healthcare. The current research shows how quality of care has changed over the years and how it correlates with changes in healthcare and health during the COVID-19-pandemic.

**Method:**

Patient experiences were collected using satisfaction ratings and the quality indicator PREM Chronic Care, among a representative group of people with a chronic disease. Trend analyses with repeated measurements (2016–2020) were performed and differences between subgroups were tested with Mann-Whitney U tests.

**Results:**

Quality of care ratings are mainly positive, also during the COVID-19-pandemic in autumn of 2020. In that year, people are the least satisfied with the coordination between healthcare providers and with preventive healthcare of their illness (64% and 67% is satisfied respectively). Trend analyses show that satisfaction with preventive healthcare has declined and satisfaction with shared decision-making has fluctuated over the years. People who experience consequences for their health(care) during the COVID-19-pandemic, are less satisfied with different aspects of healthcare quality than those who experience no consequences.

**Conclusion:**

It seems important to pay attention to patient experiences about healthcare processes, with emphasis on additional information for prevention, support for changes in health and treatment during the COVID-19-pandemic, and good coordination between healthcare providers.

## Inleiding

In de gezondheidszorg voor mensen met een chronische ziekte staat een integrale en persoonsgerichte aanpak steeds vaker centraal. Idealiter werken hierbij verschillende zorgverleners uit verschillende disciplines samen rond de zorgvraag van de patiënt [1, 2]. Zorg en ondersteuning zijn op een zodanige wijze georganiseerd dat patiënten zelf regie kunnen nemen over hun ziekte en behandeling, uitgaande van hun behoeften en waar mogelijk met ondersteuning van zorgverleners of het informele netwerk [1–3]. Een belangrijk onderdeel van zowel persoonsgerichte als geïntegreerde zorg is transparantie, namelijk het inzichtelijk maken van de kwaliteit van zorg(processen). Het uiteindelijke doel hiervan is om de kwaliteit van de zorg te verbeteren en de best passende zorg voor de patiënt te realiseren [4].

De afgelopen jaren is veel aandacht geweest voor de ontwikkeling en invoering van het meten van patiëntervaringen als kwaliteitsindicatoren in de zorg. Rapportcijfers worden bijvoorbeeld ingezet om tevredenheidsoordelen van patiënten in kaart te brengen over ontvangen gezondheidszorg of individuele zorgverleners. Daarnaast worden steeds vaker *patient reported experience measures* (PREM’s) gebruikt, vragenlijsten die ervaringen met specifieke aspecten van zorgprocessen inzichtelijk maken [4–6]. De PREM Chronische Zorg is een recentelijk ontwikkeld meetinstrument dat de kwaliteit van zorgprocessen vanuit het perspectief van mensen met een chronische ziekte meet [7]. Deze vragenlijst is gericht op de belangrijkste aspecten van de kwaliteit van de zorg en ondersteuning waarmee mensen met een chronische ziekte te maken krijgen, zoals bejegening en communicatie met zorgverleners, informatievoorziening, gezamenlijke besluitvorming en afstemming tussen verschillende zorgverleners. De PREM Chronische Zorg omvat veel aspecten van persoonsgerichte en geïntegreerde zorg, zoals gezamenlijke besluitvorming, waarbij de patiënt en betrokken zorgverlener(s) samenwerken om de meest passende zorg en behandeling te realiseren [7, 8].

De coronapandemie en de bijbehorende maatregelen hebben geleid tot veel veranderingen in de gezondheidszorg. Mensen met een chronische ziekte kregen te maken met onder andere afgeschaalde of uitgestelde zorg in het ziekenhuis, en aangepaste zorg en ondersteuning via bijvoorbeeld digitaal contact met zorgverleners. Tijdens de eerste coronagolf had een op de tien patiënten te maken met uitgestelde behandelingen en digitale afspraken [9]. Een klein deel (4%) gaf daarnaast aan zorg of ondersteuning te hebben vermeden, uit angst om besmet te raken met het coronavirus of anderen te besmetten, of doordat ze zich bezwaard voelden om contact met de zorgverlener op te nemen.

De coronapandemie heeft mogelijk ook gezorgd voor veranderingen in de gezondheid van mensen. Recent onderzoek van het Sociaal en Cultureel Planbureau laat zien dat vooral kwetsbare groepen een verslechtering in gezondheid rapporteren tijdens de coronapandemie, zoals ouderen, mensen met onderliggend lijden en personen met een lage sociaaleconomische status [10]. Ook mensen met een chronische ziekte behoren tot de kwetsbare groep die tijdens de coronapandemie geconfronteerd wordt met veel veranderingen in de organisatie en uitvoering van zorg. Uit onderzoek blijkt dat bijna een op de vijf mensen die beperkingen ervaren door hun chronische ziekte een verslechtering in gezondheid tijdens de coronapandemie rapporteert [9]. Veranderingen in zowel zorg en ondersteuning als ervaren gezondheid hebben mogelijk impact op de ervaren kwaliteit van zorg. Ze kunnen zodoende een belangrijke indicator zijn om te bepalen of bepaalde subgroepen, namelijk mensen die veranderingen in hun situatie ervaren tijdens de coronapandemie, (on)tevredener zijn over de kwaliteit van zorg en of er extra aandacht of ondersteuning voor deze subgroep zou moeten zijn.

Het huidige onderzoek maakt inzichtelijk hoe mensen met een chronische ziekte de kwaliteit van zorg ervaren tijdens de tweede coronagolf in Nederland, in het najaar van 2020, vergeleken met de situatie vóór de coronapandemie. Ook wordt gekeken in hoeverre ervaren kwaliteit van zorg samenhangt met veranderde zorg en ondersteuning, en mogelijke gezondheidsveranderingen tijdens de tweede coronagolf. De volgende onderzoeksvragen worden beantwoord:

1. Hoe beoordelen mensen met een chronische ziekte de zorg en ondersteuning verleend door verschillende zorgverleners, en de kwaliteit van de belangrijkste zorgprocessen waarmee zij te maken hebben?
2. Is er in de tweede coronagolf een verandering in de ervaren kwaliteit van zorg en ondersteuning zichtbaar onder mensen met een chronische ziekte, vergeleken met de situatie vóór de pandemie?
3. Is er een verschil in patiëntenervaringen van mensen die a) wel of geen veranderde zorg tijdens de tweede coronagolf hebben ervaren en b) wel of geen gezondheidsverandering tijdens de tweede coronagolf hebben ervaren?

## Methode

### Databron en onderzoekspopulatie

Er is gebruikgemaakt van gegevens uit het Nationaal Panel Chronisch zieken en Gehandicapten (NPCG) van het Nivel. Het NPCG is een landelijk representatief panel van ongeveer 3.500 zelfstandig wonende Nederlanders van 15 jaar en ouder. Panelleden hebben een medisch gediagnosticeerde chronische somatische ziekte en/of een lichamelijke beperking. Ze worden geworven via aselecte steekproeven van huisartsenpraktijken en bevolkingsonderzoek [3]. Leden ontvangen in het voor- en het najaar een schriftelijke of online vragenlijst over uiteenlopende thema’s rond zorg en participatie. De gegevensverzameling van het NPCG is geregistreerd bij de Autoriteit Persoonsgegevens (nr. 1283171). De gegevens worden verzameld en verwerkt in overeenstemming met de richtlijnen voor privacybescherming. Deelnemers geven schriftelijk toestemming voor deelname aan het panel.

Voor het huidige onderzoek zijn vragenlijstgegevens geselecteerd over de thema’s kwaliteit van zorg en veranderingen in zorg en gezondheid tijdens de coronapandemie. Alleen gegevens van respondenten met ten minste één chronische ziekte zijn geïncludeerd. Er is gebruikgemaakt van een longitudinaal onderzoeksontwerp met herhaalde najaarsmetingen gedurende de periode 2016–2020. Het longitudinale ontwerp is aangevuld met een cross-sectioneel onderzoeksontwerp met gegevens uit 2020, om te toetsen hoe de kwaliteit van zorg wordt beoordeeld door mensen die tijdens de coronapandemie wel of geen veranderde zorg of gezondheid ervaarden.

### Ervaren kwaliteit van zorg en ondersteuning

Ervaren kwaliteit van zorg en ondersteuning zijn in kaart gebracht via tevredenheidsoordelen over acht zorgverleners: huisarts, medisch specialist, praktijkondersteuner huisartsenzorg (POH), wijkverpleegkundige, gespecialiseerd verpleegkundige, thuiszorg, fysiotherapeut en apotheker. Aan panelleden is gevraagd om aan de zorgverlener(s) met wie het afgelopen jaar contact is geweest een rapportcijfer tussen de 0 (heel erg slecht) en 10 (uitstekend) toe te kennen. Ook is gevraagd een rapportcijfer toe te kennen aan de totale zorg die de persoon heeft ontvangen. De gegevens zijn jaarlijks beschikbaar van 2016–2020.

Daarnaast is gebruikgemaakt van de kwaliteitsindicator PREM Chronische Zorg [7]. Deze vragenlijst is een valide en betrouwbaar meetinstrument, dat rapporteert over ervaringen met verschillende aspecten van de gezondheidszorg [11]. In dit onderzoek worden ervaringen op zes domeinen gepresenteerd: bejegening, voorlichting en deskundigheid van de zorgverlener, gezamenlijke besluitvorming, preventieve begeleiding bij de ziekte door de zorgverlener, en afstemming tussen verschillende zorgverleners. Onder preventieve begeleiding wordt verstaan: het geven van advies over een gezonde leefstijl, waaronder medicatiegebruik, bewegen, roken, eten en drinken. Elk domein bestaat uit één stelling (bijvoorbeeld ‘de zorgverlener legt begrijpelijk uit’) en wordt gemeten op een vijfpuntsschaal (1 = helemaal niet mee eens tot 5 = helemaal mee eens). De gegevens zijn jaarlijks beschikbaar van 2018–2020. Gegevens van de PREM Chronische Zorg zijn niet in 2016 en 2017 verzameld. De vijfpuntsschaal is gedichotomiseerd voor de analyses naar (helemaal) ontevreden (1 = helemaal niet mee eens, 2 = oneens, 3 = niet oneens/niet eens) versus (helemaal) tevreden (4 = eens, 5 = helemaal mee eens).

### Veranderingen in zorg en ondersteuning tijdens de coronapandemie

In 2020 is aan respondenten gevraagd of de coronacrisis gevolgen heeft (gehad) voor de behandeling of professionele ondersteuning van hun chronische ziekte (antwoordoptie ‘ja/nee’). Mensen die gevolgen rapporteerden, konden aangeven welke gevolgen voor hen van toepassing waren: 1) de zorgverlener heeft een of meer behandelafspraken afgezegd; 2) de zorgverlener heeft de behandeling uitgesteld; 3) de behandeling heeft digitaal plaatsgevonden; 4) de ondersteuning thuis is verminderd of stopgezet; en 5) ik heb er zelf voor gekozen om de behandeling niet door te laten gaan.

### Veranderingen in ervaren gezondheid tijdens de coronapandemie

In 2020 is aan respondenten gevraagd of zij sinds het begin van de coronacrisis veranderingen in hun gezondheid hebben opgemerkt. De mogelijke antwoordcategorieën waren 1) nee, mijn gezondheid is niet veranderd, 2) ja, mijn gezondheid is verslechterd en 3) ja, mijn gezondheid is verbeterd.

### Achtergrondkenmerken

De volgende achtergrondkenmerken zijn verzameld: geslacht, leeftijd en opleidingsniveau (laag, middelbaar of hoog) en mate van lichamelijke beperking als gevolg van een chronische ziekte. Het type diagnose en het aantal chronische ziekten is via ICPC-codes bij huisartsen verzameld en gekoppeld aan de overige achtergrondkenmerken en de vragenlijstgegevens [3].

### Data-analyse

De statistische analyse is uitgevoerd met MLWIN 2.30 voor onderzoeksvraag 1 en 2, en StataSE 15.0 voor onderzoeksvraag 3 [12, 13]. Voor alle indicatoren voor de kwaliteit van zorg zijn trendanalyses uitgevoerd om mogelijke verschuivingen te identificeren die in de loop van de tijd hebben plaatsgevonden. Voor de beoordelingscijfers van de totale zorg en de individuele zorgverleners zijn gegevens van vijf jaar (2016–2020) bekeken. Voor de kwaliteitsindicator PREM zijn gegevens uit drie jaar bekeken (2018–2020). Hiervoor is een multilevelmodel gebruikt met geneste waarnemingen voor de herhaalde metingen. De resultaten van de multilevelanalyses zijn schattingen van de gemiddelde waarden of percentages met een 95%-betrouwbaarheidsinterval (95%-BI). De schattingen zijn gebaseerd op een standaardpopulatie van mensen met een chronische ziekte (zie digitaal aanvullende content, tabel 1). Door de standaardisatie wordt een vertekening van resultaten over de tijd voorkomen en kunnen de uitkomsten van verschillende meetmomenten met elkaar vergeleken worden. De standaardpopulatie is samengesteld op basis van alle nieuwe instromers met een chronische ziekte in het NPCG tussen 2010 en 2015. Verschillen tussen mensen die wel of geen veranderingen in zorg of gezondheid gedurende de coronapandemie hebben ervaren, zijn getoetst met Mann-Whitney U‑toetsen (niet gestandaardiseerd, najaar van 2020). Een *p*-waarde kleiner dan 0,01 is statistisch significant. Ontbrekende waarden zijn niet meegenomen in de analyses.

## Resultaten

### Steekproefbeschrijving

Binnen de vijf metingen (2016–2020) liep het aantal respondenten uiteen van 1.197 tot 1.512 personen. De respons was gemiddeld 77% (zie digitaal aanvullende content, tabel 2). In het meetjaar 2020 was iets meer dan de helft van de respondenten vrouw (54,6%). De gemiddelde leeftijd was 66 jaar, waarbij de leeftijdscategorie van 65 tot en met 74 jaar het meest vertegenwoordigd was. Ongeveer een vierde van de respondenten had een laag opleidingsniveau, 29,1% had een hoge opleiding afgerond. Veel voorkomende chronische ziekten waren hart- en vaatziekten (21,4%), diabetes (15,4%) en astma of COPD (15,4%). Drieënvijftig procent van de respondenten had één chronische ziekte, 18,5% had er drie of meer. Het merendeel had geen of lichte lichamelijke beperkingen (71,4%). Zie digitaal aanvullende content, tabel 1 voor alle achtergrondkenmerken.

### Beoordelingscijfers totale zorg en zorgverleners

**Figure 1:**
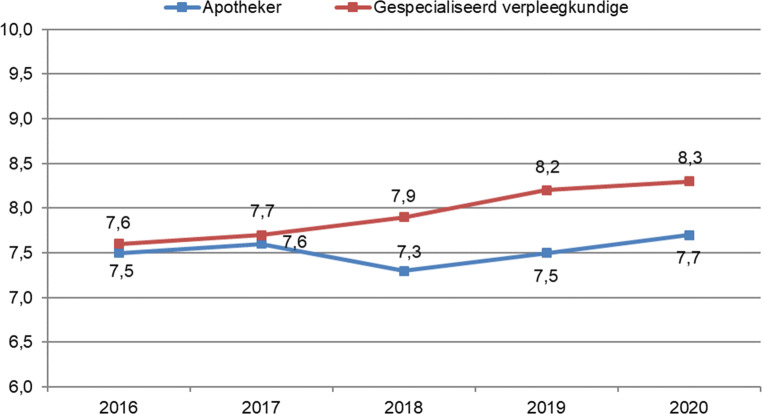

In 2020 beoordeelden mensen met een chronische ziekte de totale zorg die zij ontvingen als goed, met een gemiddeld rapportcijfer van 7,8. Het cijfer was constant gedurende de periode 2016–2020. De gespecialiseerd verpleegkundige ontving de hoogste beoordeling in 2020 (8,3; 95%-BI 8,0–8,5). Het gemiddelde cijfer van de gespecialiseerd verpleegkundige is toegenomen over de jaren (zie fig. 1; lineaire trend). De apotheker werd het laagst beoordeeld met een rapportcijfer van 7,7 in 2020 (95%-BI 7,5–7,8). Het beoordelingscijfer schommelt door de jaren heen (zie fig. 1; kwadratische trend).

De gemiddelde rapportcijfers voor de thuiszorg, de fysiotherapeut en de medisch specialist zijn door de jaren heen toegenomen. In 2016 gaven mensen de thuiszorg nog een rapportcijfer van 6,6 (95%-BI 5,9–7,3). De jaren daarna werd de thuiszorg steeds beter beoordeeld, waarbij een 8,2 (95%-BI 7,6–8,8) werd toegekend in 2020 (lineaire trend). Ook de rapportcijfers voor de fysiotherapeut en de medisch specialist stegen in 2020 respectievelijk naar een 8,2 (95%-BI 8,0–8,3) en 8,0 (95%-BI 7,9–8,2) (lineaire trends). De POH werd beoordeeld met een 7,8 gemiddeld over de periode 2016–2020. Het beoordelingscijfer schommelt door de jaren heen (polynomiale trend). Er zijn geen veranderingen zichtbaar in de beoordeling van de huisarts en de wijkverpleegkundige. In 2020 ontvingen zij respectievelijk een 8,0 (95%-BI 7,9–8,2) en 7,9 (95%-BI 6,8–9,0) gemiddeld. Zie digitaal aanvullende content, tabel 3 voor alle beoordelingscijfers per meetmoment.

### Kwaliteitsindicator PREM

In 2020 gaf de grote meerderheid van mensen met een chronische ziekte aan tevreden te zijn met zowel de bejegening (95,4%) en de voorlichting (95,0%), als de deskundigheid van de zorgverlener (92,1%; zie tab. 1). Minder mensen waren tevreden met de afstemming tussen zorgverleners, namelijk 64,0%. De beoordelingen van deze vier domeinen blijft door de jaren heen constant. Bij de kwaliteitsindicatoren preventieve begeleiding en gezamenlijke besluitvorming zijn veranderingen in de tijd zichtbaar. Het percentage mensen dat tevreden is over de preventieve begeleiding van hun ziekte nam af tot 67,1% in 2020. Voor de jaren 2018 en 2019 was dit percentage nog respectievelijk 79,3% (95%-BI 74,8–83,2) en 76,8% (95%-BI 72,6–80,6). Over de jaren heen was er een wisselende tevredenheid zichtbaar over de gezamenlijke besluitvorming. Zo daalde het percentage mensen dat aangaf samen met hun zorgverleners over hun behandeldoelen te spreken van 72,4% in 2018 (95%-BI 67,0–77,2) naar 65,1% (95%-BI 59,5–70,3) in 2019. Vervolgens steeg dit aantal naar 84,5% (95%-BI 80,2–88,0) in 2020. Zie digitaal aanvullende content, tabel 4 voor alle beoordelingscijfers per meetmoment.

**Table 1:** 

Domein	Totale *n* ^a^	% (helemaal) tevreden (95%-BI)	Trend, 2018–2020
Bejegening	778	95,4 (93,0–97,2)	Geen
Voorlichting	772	95,0 (92,0–96,4)	Geen
Deskundigheid	775	92,1 (89,0–95,0)	Geen
Gezamenlijke besluitvorming	777	84,5 (80,2–88,0)	Lineair (stijgend) en kwadratisch
Preventieve begeleiding	774	67,1 (62,0–72,0)	Lineair (dalend)
Afstemming zorgverleners	773	64,0 (59,0–69,1)	Geen

De scores zijn gedichotomiseerd weergegeven (helemaal oneens, oneens of niet oneens/niet eens versus eens of helemaal eens). Gewogen gegevens

^a^Het betreft het aantal cases zonder ontbrekende waarden. Hier zitten ook mensen bij die (helemaal) oneens of niet oneens/niet eens hebben geantwoord. Alleen complete cases zijn meegenomen in de analyse. Ontbrekende waarden zijn niet meegenomen

### Kwaliteit van zorg en veranderingen in zorg en gezondheid tijdens de coronapandemie

Een vierde van de mensen met een chronische ziekte (25,3%, *n* = 295) had in het najaar van 2020 te maken met een of meer veranderingen in de professionele zorg of ondersteuning. De meest gerapporteerde veranderingen waren behandelafspraken die digitaal plaatsvonden (11,0%, *n* = 128), afgezegde behandelafspraken (10,6%, *n* = 124) en uitgestelde behandelafspraken (8,2%, *n* = 95). De ervaren kwaliteit van zorg verschilt tussen mensen die wel of geen veranderingen hebben ondervonden voor hun behandeling of ondersteuning (zie tab. 2). Diegenen die geen veranderingen ondervonden beoordeelden de zorg met een hoger cijfer (*p* < 0,001). Zo gaven zij vaker het hoogst mogelijke cijfer in vergelijking met diegenen die tijdens de coronacrisis wel veranderingen hebben gemerkt (16,7% versus 6,6%). Ook rapporteerde de groep die veranderingen in de zorg heeft ervaren minder vaak dat er preventieve begeleiding plaatsvond (58,9%), dan de groep die geen veranderde zorg of ondersteuning heeft ervaren (67,6%; *p* = 0,003). De twee groepen verschillen daarnaast in hun kwaliteitsoordeel over de afstemming tussen zorgverleners. Van de mensen die geen veranderingen hebben ervaren vond 61% de afstemming tussen hun zorgverleners goed, in tegenstelling tot 51,1% van de mensen die wel gevolgen hebben ervaren (*p* = 0,002).

**Table 2:** 

	Veranderde zorg of ondersteuning tijdens de coronacrisis^a^					Mann-Whitney U
	Ja		Nee			
	*n*	%	*n*	%	Totale *n* ^b^	*p*-waarde
**Cijfer totale zorg**	-	-	-	-	1.017	-
< 6	15	5,5	51	6,9	-	< 0,001
6	14	5,1	24	3,2	-	-
7	61	22,3	116	15,6	-	-
8	126	46,2	294	39,5	-	-
9	39	14,3	135	18,1	-	-
10	18	6,6	124	16,7	-	-
**Bejegening** (helemaal) tevreden	221	94,4	555	93,4	828	0,695
**Voorlichting** (helemaal) tevreden	217	93,9	552	93,4	822	0,993
**Deskundigheid** (helemaal) tevreden	210	90,5	534	90,4	823	0,706
**Gezamenlijke besluitvorming** (helemaal) tevreden	187	80,3	490	82,6	826	0,236
**Preventieve begeleiding **(helemaal) tevreden	136	58,9	401	67,6	824	0,003
**Afstemming zorgverleners** (helemaal) tevreden	119	51,1	360	61,0	823	0,002

^a^Er is gebruikgemaakt van de dichotome variabele ‘ja’/‘nee’ ervaren verandering in zorg of ondersteuning

^b^Het betreft het aantal cases zonder ontbrekende waarden. Voor de PREM-kwaliteitsindicator zitten hier ook mensen bij die (helemaal) oneens of niet oneens/niet eens hebben geantwoord

In een subanalyse is gekeken of er verschil is in beoordelingscijfers en kwaliteitsoordelen tussen mensen die wel of niet te maken kregen met a) afgezegde behandelafspraken, b) uitgestelde behandelafspraken en c) digitale behandelafspraken (data niet weergegeven). Diegenen die te maken kregen met afgezegde behandelafspraken beoordeelden de zorg met een lager cijfer en waren iets minder vaker tevreden over de preventieve begeleiding (*p* < 0,05). Mensen die te maken kregen met uitgestelde of digitale behandelafspraken waren iets minder vaak tevreden over de afstemming tussen zorgverleners (*p* < 0,05).

De meerderheid van de mensen met een chronische ziekte ervaarde in het najaar van 2020 geen verandering in gezondheid (83,9%, *n* = 967). Circa een op de zes rapporteerde een verslechtering in gezondheid (14,4%, *n* = 166) en een kleine minderheid rapporteerde een verbetering (1,7%, *n* = 20). Gezondheidsveranderingen hangen samen met ervaren kwaliteit van zorg (zie tab. 3). Van de mensen die een verslechtering in gezondheid hebben ervaren, beoordeelde 6,7% de totale zorg met het hoogst mogelijke cijfer, in vergelijking met 15,2% van de mensen die geen verslechtering hebben ervaren (*p* < 0,001). De twee groepen beoordeelden daarnaast de mate van afstemming tussen zorgverleners verschillend. Van de mensen die een verslechtering in gezondheid heeft ervaren, is 44,0% tevreden met de afstemming tussen zorgverleners, in tegenstelling tot 60,5% van de mensen die geen verslechtering in gezondheid rapporteerde (*p* = 0,001).

**Table 3:** 

	Verslechterde gezondheid tijdens de coronacrisis^a^					Mann-Whitney U
	Ja		Nee		Totale *n* ^b^	
	*n*	%	*n*	%		*p*-waarde
**Cijfer totale zorg**	-	-	-	-	986	-
< 6	15	10,1	48	5,7	-	< 0,001
6	11	7,4	29	3,5	-	-
7	29	19,5	139	16,6	-	-
8	68	45,6	339	40,5	-	-
9	16	10,7	155	18,5	-	-
10	10	6,7	127	15,2	-	-
**Bejegening** (helemaal) tevreden	103	88,0	642	94,7	795	0,994
**Voorlichting** (helemaal) tevreden	109	93,2	629	93,5	790	0,413
**Deskundigheid** (helemaal) tevreden	97	83,6	620	91,7	792	0,051
**Gezamenlijke besluitvorming** (helemaal) tevreden	86	74,1	563	83,0	794	0,038
**Preventieve begeleiding **(helemaal) tevreden	67	57,3	448	66,4	792	0,094
**Afstemming zorgverleners** (helemaal) tevreden	51	44,0	408	60,5	790	0,001

^a^Door (te) kleine aantallen (*n* = 20) is de groep die rapporteerde een verbetering in gezondheid te hebben ervaren niet meegenomen. Er is gebruikgemaakt van de dichotome variabele ‘wel’/‘geen’ verslechtering in gezondheid

^b^Het betreft het aantal cases zonder ontbrekende waarden. Voor de PREM-kwaliteitsindicator zitten hier ook mensen bij die (helemaal) oneens of niet oneens/niet eens hebben geantwoord

## Beschouwing en conclusie

Het huidige onderzoek onder een representatieve groep van mensen met een chronische ziekte in Nederland laat zien dat de kwaliteit van de zorg en ondersteuning over het algemeen positief wordt ervaren, ook tijdens de coronapandemie in het najaar van 2020. Mensen met een chronische ziekte beoordelen de totale zorg die zij ontvangen in 2020 als goed, met een gemiddeld rapportcijfer van 7,8. Ook de tevredenheidscijfers voor individuele zorgverlener zijn over het algemeen hoog. Voor een deel van de zorgverleners, zoals de huisarts en wijkverpleegkundige, zijn de beoordelingen in de periode 2016 tot 2020 niet veranderd. Voor een ander deel van de zorgverleners, zoals de gespecialiseerd verpleegkundige en de medisch specialist, zetten de stijgende trends in beoordelingscijfers die sinds 2016 zichtbaar zijn, door.

Voor een subgroep van de mensen lijken de coronapandemie en de bijbehorende maatregelen in 2020 invloed te hebben op hun ervaren kwaliteit van zorg. Mensen die te maken hebben met uitgestelde of veranderde zorg en ondersteuning beoordelen de zorg minder positief, dan mensen die geen gevolgen ervaren. Ook de groep die tijdens het najaar van 2020 een verslechtering in de gezondheidstoestand ervaart, beoordeelt de zorg minder positief. Het is mogelijk dat mensen door veranderingen in de zorg en gezondheid tijdens de coronapandemie minder tevreden zijn met de kwaliteit van de zorg. Het is ook mogelijk dat degenen die de kwaliteit van de zorg lager beoordelen al vóór de coronapandemie meer gebruikmaakten van zorg en ondersteuning, waardoor ze extra geraakt zijn door de pandemie en -maatregelen. In hoeverre veranderingen in zorg en ondersteuning, gezondheidsveranderingen en de ervaren kwaliteit van zorg op elkaar inspelen behoeft vervolgonderzoek. Desondanks is het belangrijk om aandacht te schenken aan de mogelijke impact van de coronapandemie op iemands gezondheid en behandeling. Twee van de vijf mensen met een chronische ziekte geven in eerder onderzoek aan dat uitgestelde of verschoven zorg gevolgen voor ze heeft [9]. Ander onderzoek laat daarnaast zien dat ruim 35% van de mensen met een langdurige ziekte bang is dat hun bestaande klachten verergeren of onopgemerkt blijven door de maatregelen rondom de pandemie [14]. Dit komt overeen met bevindingen van Duits onderzoek, dat onder andere rapporteert dat mensen met een chronische ziekte tijdens de coronapandemie bezorgd zijn over ontoereikende of vertraagde behandelingen, tekorten aan medicatie of aanpassingen in zelfmanagement door de veranderde dagelijkse omstandigheden als gevolg van de coronamaatregelen [15].

Zorgprocessen worden over het algemeen goed beoordeeld door mensen met een chronische ziekte. Dit komt overeen met eerdere onderzoeksbevindingen met het huidige panel [3]. Internationaal onderzoek laat zien dat veel patiënten tevreden zijn met de deskundigheid van professionals en zich door hen gesteund voelen bij de behandeling van hun ziekte [16]. Als we kijken naar veranderingen in de tijd zien we in het huidige onderzoek een opvallende schommeling bij de tevredenheid over gezamenlijke besluitvorming, waarbij het percentage mensen dat tevreden is in 2019 eerst daalt en daarna in 2020 stijgt. Het is onduidelijk wat de verklaring voor deze stijging is en in hoeverre de hogere tevredenheid wel of niet gerelateerd is aan het veranderde zorglandschap tijdens de coronapandemie. De pandemie heeft naast uitdagingen ook kansen gecreëerd voor het leveren van zorg. Het gebruik van digitale toepassingen kan bijvoorbeeld zorgen voor een meer gepersonaliseerde context voor gezamenlijke besluitvorming [17]. Het is ook mogelijk dat mensen met een chronische ziekte sinds de pandemie meer regie over hun behandeling nemen en meer inspraak willen, bijvoorbeeld omdat ze zich door het virus bewuster zijn van de kwetsbaarheid van hun gezondheid.

Ongeveer een derde van de mensen met een chronische ziekte is niet tevreden over de preventieve begeleiding voor hun ziekte. De tevredenheidscijfers laten hierbij een duidelijk dalende trend zien. Ook hier zou de coronapandemie een mogelijke verklaring voor de cijfers kunnen vormen. Het belang van een gezonde voeding en voldoende beweging, die onderdeel zijn van de preventie, zijn sinds de start van de coronapandemie benadrukt [18]. De resultaten van onderzoeken die veranderingen in leefstijl door corona in kaart brengen zijn wisselend. Zo blijkt uit vragenlijstonderzoek onder een grote steekproef van de Italiaanse bevolking dat de fysieke activiteit gedurende de pandemie iets is toegenomen [19], terwijl veel jongeren met een chronische aandoening in Nederlands onderzoek aangeven het moeilijk te vinden om gezonde keuzen te maken tijdens de coronacrisis [14]. Het is mogelijk dat mensen met een chronische ziekte zich bewuster zijn van het belang van een gezonde leefstijl en hier meer begeleiding in willen dan vóór de coronapandemie, maar het kan ook zo zijn dat mensen juist door de aandacht voor leefstijl strenger zijn in hun beoordeling dan in eerdere jaren. In hoeverre deze trend zich de komende jaren verder ontwikkelt, moet worden gemonitord.

Mensen met een chronische ziekte zijn het minst tevreden over de afstemming tussen zorgverleners; 36% van hen is niet tevreden. Het percentage is nog groter in de groep mensen die ervaren dat de coronacrisis gevolgen heeft voor hun zorg en ondersteuning, en hun gezondheid. De tevredenheid over de afstemming tussen zorgverleners is in 2020 niet anders dan in eerdere jaren. Al sinds enige jaren blijkt uit zowel nationaal als internationaal onderzoek dat een grote groep patiënten ontevreden is over de afstemming tussen zorgverleners en de toegang tot multidisciplinaire zorg [3, 20, 21]. Een op de vier mensen met een chronische ziekte geeft bijvoorbeeld aan dat zorgverleners zeer beperkt met elkaar overleggen over hun zorg [3]. Tijdens de coronapandemie zijn veel regionale en nationale samenwerkingsverbanden tussen zorgorganisaties opgezet [22, 23], waarbij de lessen en best practices zouden kunnen dienen om integraal beleid voor mensen met een chronische ziekte verder te verbeteren.

Het huidige onderzoek kent sterke punten, zoals het longitudinale karakter met meerdere meetjaren. Hierdoor is het mogelijk om uitspraken te doen over de ontwikkeling in ervaren kwaliteit van zorg en konden we in kaart brengen of een bepaalde trend die vóór de coronapandemie zichtbaar werd tijdens het najaar van 2020 al dan niet verder doorzette. Door gebruik te maken van een groot panel met aselecte wervingsmethoden en wegingen zijn de uitkomsten van het onderzoek daarnaast generaliseerbaar naar de groep mensen met een chronische ziekte in Nederland.

Het onderzoek kent ook beperkingen. Ongeveer een derde van de PREM-items bestond uit ontbrekende waarden. In het laatste meetjaar waren dit relatief meer ontbrekende waarden op de domeinen bejegening, voorlichting en deskundigheid dan in eerdere jaren. Op de domeinen gezamenlijke besluitvorming, preventieve begeleiding en afstemming zien we door de jaren heen schommelende ontbrekende waarden, maar in het algemeen relatief meer ontbrekende waarden dan op de andere drie domeinen. Het is belangrijk om te bedenken dat sommige stellingen van dit meetinstrument wellicht niet begrijpelijk waren of niet van toepassing waren op de situatie van een deel van de respondenten, bijvoorbeeld omdat ze niet te maken hadden met verschillende zorgverleners die zorg moesten afstemmen. De uiteindelijke aantallen waren echter voldoende om uitspraken over deze kwaliteitsindicator te doen. Tevens kunnen veranderingen in ervaren kwaliteit van zorg niet met zekerheid toegeschreven worden aan de coronapandemie. Aanvullend onderzoek is nodig om de effecten van de pandemie op de ervaren kwaliteit van zorg te specificeren en in context te plaatsen, bijvoorbeeld door kwalitatief onderzoek of door monitoronderzoek om te volgen of en hoe de huidige trends zich in de toekomst voortzetten.

Het onderzoek laat zien dat mensen met een chronische ziekte over het algemeen positief zijn over de kwaliteit van de zorg in Nederland, ook tijdens de coronapandemie. Over sommige kwaliteitsprocessen is men minder tevreden, zoals de afstemming tussen zorgverleners en de preventieve begeleiding bij de ziekte. Er is een afname zichtbaar in de tevredenheid over adviezen over preventie die mensen krijgen. Veel mensen met een chronische ziekte hebben tijdens de coronapandemie te maken gekregen met veranderingen in de gezondheidszorg. Het lijkt belangrijk om aandacht te schenken aan patiëntervaringen met zorgprocessen, waarbij extra nadruk zou moeten liggen op informatie over preventie, ondersteuning bij veranderingen in de gezondheid en behandeling tijdens de coronapandemie en goede afstemming tussen zorgverleners.
