# Supplementary material for: Positive assessment of care has changed little during the COVID-19 pandemic, but there is still room for improvement
Source: TSG. 2022 Jan 26;100(1):1–8. [Article in Dutch] doi: 10.1007/s12508-022-00329-y (PMC8790947; doi:10.1007/s12508-022-00329-y)
Supplement: Supplementary file 1 [file 12508_2022_329_MOESM1_ESM.docx]

Bijlage

Tabel 1. Kenmerken van de standaardpopulatie en respondenten van het NPCG in 2020 (%)

|  | Respondenten NPCG najaarsmeting 2020 | Standaardpopulatie van mensen met een chronische ziekte |
| --- | --- | --- |
| *N* | 1.220 | 4.076 |
| **Geslacht** |  |  |
| Man | 45,4 | 43,4 |
| Vrouw | 54,6 | 56,6 |
| **Leeftijd** |  |  |
| 15 t/m 39 jaar | 4,8 | 15,2 |
| 40 t/m 64 jaar | 32,9 | 37,0 |
| 65 t/m 74 jaar | 36,2 | 27,0 |
| 75 jaar en ouder | 26,2 | 20,8 |
| Opleidingsniveau |  |  |
| Laag (t/m lbo) | 26,1 | 32,7 |
| Midden (voortgezet onderwijs, mbo) | 44,9 | 44,4 |
| Hoog (hbo, wo) | 29,1 | 22,8 |
| Diagnose (indexziekte)^a^ |  |  |
| Diabetes | 15,4 | 13,8 |
| Astma of COPD | 15,4 | 20,0 |
| Chronische ziekte bewegingsapparaat | 11,4 | 10,7 |
| Kanker | 7,0 | 5,3 |
| Hart- en vaatziekten | 21,4 | 21,2 |
| Neurologische ziekte | 7,4 | 6,9 |
| Chronische spijsverteringsziekte | 3,7 | 5,5 |
| Andere chronische ziekte | 18,4 | 16,6 |
| Aantal chronische aandoeningen |  |  |
| Eén | 53,0 | 44,9 |
| Twee | 28,5 | 30,4 |
| Drie of meer | 18,5 | 24,7 |
| Mate van beperking |  |  |
| Geen of lichte lichamelijke beperking | 71,4 | 66,7 |
| Matige of ernstige lichamelijke beperking | 28,6 | 33,4 |

Vanwege afronding bedraagt de som van de percentages mogelijk niet 100%.

^a^ De indexziekte is in geval van de aanwezigheid van meerdere chronische ziekten de eerst gediagnosticeerde ziekte, dus de ‘oudste’ chronische ziekte.

**Tabel 2. Respons per meetjaar**

| **Meetjaar** | **Vragenlijsten verstuurd (N)** | **Vragenlijsten ingevuld (N)** | **Respons (%)** |
| --- | --- | --- | --- |
| 2016 | 1.911 | 1.512 | 79,1 |
| 2017 | 1.515 | 1.197 | 79,0 |
| 2018 | 1.610 | 1.210 | 75,2 |
| 2019 | 1.924 | 1.422 | 73,9 |
| 2020 | 1.564 | 1.220 | 78,0 |

Tabel 3. Beoordeling totale zorg en individuele zorgverleners, spreiding 0-10. Gemiddelde per meetjaar en trends (2016-2020)

|  | Gemiddelde (95%-BI; n) | | | | | Trend |
| --- | --- | --- | --- | --- | --- | --- |
|  | **2016** | **2017** | **2018** | **2019** | **2020** | **2016-2020** |
| Totale ontvangen zorg | 7,8  (7,7-7,9; 1.281) | 7,8  (7,7-7,9; 1.016) | 7,8  (7,7- 7,9; 1.046) | 7,9  (7,8- 8,0; 1.224) | 7,8  (7,7-7,9; 1.010) | n.s. |
| Gespecialiseerd verpleegkundige | 7,6  (7,4-7,8; 369) | 7,7  (7,5-7,9; 266) | 7,9  (7,6-8,1; 293) | 8,2  (8,1-8,4; 362) | 8,3  (8,0-8,5; 267) | p < 0,001 (lineair) |
| Apotheker | 7,5  (7,4-7,6; 1.155) | 7,6  (7,4-7,7; 867) | 7,3  (7,2-7,5; 929) | 7,5  (7,3-7,6; 1.042) | 7,7  (7,5-7,8; 898) | p < 0,01 (kwadratisch) |
| Thuiszorg | 6,6  (5,9-7,3; 65) | 7,4  (6,8-8,0; 103) | 7,3  (6,7-7,9; 94) | 7,9  (7,4-8,5; 116) | 8,2  (7,6-8,8; 89) | p < 0,001 (lineair) |
| Fysiotherapeut | 7,9  (7,8-8,1; 566) | 7,9  (7,8-8,1; 440) | 8,1  (7,9-8,2; 479) | 8,3  (8,1-8,4; 526) | 8,2  (8,0-8,3; 442) | p < 0,01 (lineair) |
| Medisch specialist | 7,7  (7,6-7,8; 897) | 7,8  (7,6-7,9; 656) | 7,8  (7,6-7,9; 686) | 8,0  (7,9-8,2; 834) | 8,0  (7,9-8,2; 658) | p < 0,001 (lineair) |
| Praktijkondersteuner huisarts | 7,8  (7,7-8,0; 726) | 7,7  (7,5-7,8; 569) | 7,7  (7,6-7,9; 631) | 8,0  (7,8-8,2; 703) | 7,8  (7,6-8,0; 629) | p < 0,01 (polynomiaal) |
| Huisarts | 7,9  (7,8-8,0; 1.186) | 7,9  (7,8-8,0; 889) | 7,8  (7,6-7,9; 954) | 8,0  (7,9-8,2; 1.068) | 8,0  (7,9-8,2; 896) | n.s. |
| Wijkverpleegkundige | 7,4  (6,9-8,0; 1.259) | 7,6  (6,9-8,2; 917) | 7,6  (6,9-8,4; 954) | 7,7  (6,9-8,5; 1.064) | 7,9  (6,8-9,0; 935) | n.s. |

Rapportcijfers worden alleen gegeven indien respondenten de afgelopen 12 maanden contact hebben gehad met de betreffende zorgverlener. Gewogen gegevens. 95%-BI = 95%-betrouwbaarheidsinterval; n.s.= niet significante trend. Lineaire trend = er is sprake van een geleidelijke toe- of afname in tijd. Kwadratische trend = er is sprake van een toename, gevolgd door een afname, of andersom. Polynomiale trend = de waarden fluctueren over de tijd.

**Tabel 4. Kwaliteitsindicator PREM Chronische Zorg. Percentages per meetjaar en trends (2018, 2019 en 2020)**

|  | % (helemaal) tevreden (95%-BI, n) | | | Trend |
| --- | --- | --- | --- | --- |
| Domein | **2018** | **2019** | **2020** | **2018-2020** |
| Bejegening, (helemaal) tevreden | 95,3  (92,6-97,1; 927) | 95,5  (93,3-97,0; 1027) | 95,4  (93,0-97,2; 778) | n.s. |
| Voorlichting, (helemaal) tevreden | 93,6  (90,8-95,6; 910) | 94,4  (91,8-96,1; 1.014) | 95,0  (92,0-96,4; 772) | n.s. |
| Deskundigheid, (helemaal) tevreden | 91,3  (88,0-93,8; 910) | 93,2  (90,5-95,2; 1.030) | 92,1  (89,0-95,0; 775) | n.s. |
| Gezamenlijke besluitvorming, (helemaal) tevreden | 72,4  (67,0-77,2; 617) | 65,1  (59,5-70,3; 629) | 84,5  (80,2-88,0; 777) | p < 0,001  (lineair en kwadratisch |
| Preventieve begeleiding, (helemaal) tevreden | 79,3  (74,8-83,2; 760) | 76,8  (72,6-80,6; 834) | 67,1  (62,0-72,0; 774) | p < 0,001  (lineair) |
| Afstemming zorgverleners, (helemaal) tevreden | 68,5  (63,6-73,1; 777) | 70,7  (66,0-75,1; 767) | 64,0  (59,0-69,1; 773) | n.s. |

Elk domein van de PREM Chronische Zorg bestaat uit stellingen die gescoord worden op een vijfpuntschaal (helemaal oneens tot helemaal eens) en een ‘niet van toepassing’-categorie. De scores zijn gedichotomiseerd weergegeven (helemaal oneens, oneens of niet oneens/niet eens; eens of helemaal eens). Gewogen gegevens. 95%-BI = 95%-betrouwbaarheidsinterval; n.s.= niet significante trend. Lineaire trend = er is sprake van een geleidelijke toe- of afname in tijd. Kwadratische trend = er is sprake van een toename, gevolgd door een afname, of andersom. Polynomiale trend = de waarden fluctueren over de tijd.
